# Lower Incidence of Hypo-Magnesemia in Surgical Intensive Care Unit Patients in 2011 Versus 2001

**DOI:** 10.14740/jocmr2101w

**Published:** 2015-02-09

**Authors:** John T. Denny, Enrique Pantin, Antonio Chiricolo, James Tse, Thomas Jan, Mohammad Chaudhry, Sylviana Barsoum, Angela M. Denny, Denes Papp, Sharon L. Morgan

**Affiliations:** a Rutgers Robert Wood Johnson Medical School, 3100 CAB, 125 Paterson Street, New Brunswick, NJ 08901, USA; b Rutgers Graduate School of Nursing, 65 Bergen Street, Newark, NJ 07107, USA

**Keywords:** Magnesium, Hypo-magnesemia, Divalents in ICU, Electrolyte abnormalities

## Abstract

**Background:**

Hypo-magnesemia is described to occur in as many as 65% of intensive care unit (ICU) patients. Magnesium (Mg) is a cofactor in over 300 enzymatic reactions involving energy metabolism, protein, and nucleic acid synthesis. The membrane pump that creates the electrical gradient across the cell membrane is dependent on Mg, and it is important in the activity of electrically excitable tissues. Since Mg regulates the movement of calcium in smooth muscle cells, it is also important in peripheral vascular tone and blood pressure. Studies have linked hypo-magnesemia to multiple chronic diseases and to a higher mortality rate.

**Methods:**

To explore trends within our own tertiary care surgical ICU, we sampled our patients’ laboratory records in 2001 and in 2011. Hypo-magnesemia in our ICU is defined as an Mg less than 2.0 mg/dL.

**Results:**

This retrospective review of all SICU patients from October to December revealed that there was a significant increase (P < 0.01) in the patients with their serum Mg level measured between 2001 (89%) and 2011 (95%). There was a significant decrease (P < 0.001) in patients with hypomagnesemia (< 2 mg/dL) between 2001 (47.5%) and 2011 (33.0%). On the other hand, there was a significant increase (P < 0.001) in patients with normal serum Mg level (> 2 mg/dL) between 2001 (52.5%) and 2011 (67.0%).

**Conclusions:**

There was not only more monitoring of Mg in 2011, but a lower incidence of hypo-Mg compared to 2001. Possible explanations include changing patterns of antibiotic and diuretic use, less amphotericin use, more frequent laboratory surveillance, and better trained ICU practitioners.

## Introduction

Hypo-magnesemia is a common laboratory abnormality encountered in hospitalized patients. It is found in as many as 20% of medical floor patients [1]. It has been described to occur in as many as 65% of patients in intensive care units (ICUs) [2]. Magnesium (Mg)’s importance as an essential nutrient has been realized since Kruse in 1932 described the effects of acute Mg deficiency in rats [3]. It is the second most plentiful intracellular cation, and the fourth most occurring cation in the body [4]. The physiologically active form of the element is ionized. Protein-bound and chelated Mg buffer the ionized pool. Approximately 50% of the total Mg in the body is present intracellularly in soft tissue, while the other half is present in bone. Only 0.3% of total body Mg is present in the serum [2], while less than 1% of the total body Mg is present in blood [4]. Estimates of Mg deficiency range from 20% to 65% [2, 5, 6]. Rubeiz et al found that reductions in total serum Mg found at hospital admission are associated with increased mortality [7]. Sakaguchi et al found that hypomagnesemia was significantly associated with an increased risk of mortality in hemodialysis patients [8]. Guasch-Ferre et al reported an inverse relationship between dietary Mg ingestion and mortality in patients with elevated cardiovascular risk [9].

## Methods

Our 17-bed trauma/surgical ICU (SICU) is part of a 600-bed tertiary care hospital that is a designated level one trauma center. The SICU admits patients with surgical issues, and trauma patients. Patient age ranges from 21 to 105 years old. Patients younger than 21 years old are admitted to the pediatric ICU at our institution. Critically ill patients get laboratory studies drawn at 1 am each day. If conditions warrant, they often have repeat labs done throughout the day. We reviewed patient records for 2001 and 2011. All lab values in the SICU during this time were recorded. The last 3 months of each year were selected to compare similar times. Mg levels less than 2.0 were below our normal ICU range and were noted.

## Results

This retrospective review shows that there were 67 SICU patients in 2001 and 50 SICU patients in 2011 during October to December (Table 1). There was similar ratio of male vs. female patients and average age of SICU patients during these periods. The diagnosis and surgical procedures included: s/p open abdominal aortic aneurysm (AAA) repair, s/p perforated duodenal ulcer, status epilepticus, s/p carotid endarterectomy, liver laceration, pancreatic abcess, renal laceration, liver cancer, colon perforation, rib fractures/hemothorax, DVT, subarachnoid hemorrhage/hydrocephalus, facial fractures, pituitary tumor, subarachnoid hemorrhage/vasospasm, s/p radical cystectomy, s/p small bowel resection, s/p perforated diverticulitis, brain tumor, closed head injury, s/p excision of pelvic mass, subdural hematoma, pelvic fracture, femur fracture, cholangitis, intracranial hemorrhage, bowel obstruction, foot gangrene/PVD, splenectomy, splenic laceration, enterocutaneous fistula, subarachnoid hemorrhage, spinal cord injury, cerebral aneurysm, spine fracture, skull fracture, pancreatitis, hemorrhagic cystitis, brain metastasis, colon cancer, renal cell cancer, pancreatic cancer, spinal stenosis, spinal tumor, bowel ischemia, gunshot wound to chest, pancreas renal transplant, thoracic aneurysm, ARDS, s/p endovascular AAA, esophageal perforation, s/p esophageal cancer, rib fractures, rectal cancer, uterine bleeding, infected vascular graft, pulmonary embolus, pseudoaneurysm, and s/p brain stimulator.

**Table 1:** Sex and Average Age of SICU Patients During October to December in 2001 and 2011

October - December	Number of SICU patients	Sex	Average age (years)
2001	67	48.9% male	61.4
2011	50	52.2% male	66.2

The total number of electrolyte determinations was 314 in 2001 and 296 in 2011. The average number of electrolyte determination was 4.69 per patient in 2001 and 5.92 per patient during October to December. There was a significant increase (P < 0.01) in electrolyte determinations with serum Mg level measured between 2001 (89%) and 2011 (95%) (Table 2). There was a significant decrease (P < 0.001) in electrolyte determinations with hypomagnesemia (< 2 mg/dL) between 2001 (47.5%) and 2011 (33.0%) (Table 2). On the other hand, there was a significant increase (P < 0.001) in electrolyte determinations with normal serum Mg level (> 2 mg/dL) between 2001 (52.5%) and 2011 (67.0%) (Table 2).

**Table 2:** Comparing Serum Magnesium Levels in SICU Patients During October to December During 2001 and 2011

	Number of SICU patients during October - December	Total electrolyte determinations during October - December	Electrolyte determinations with serum Mg level measured	Electrolyte determinations with hypo-Mg level (< 2 mg/dL)	Electrolyte determination with normal Mg level (> 2 mg/dL)
2001 (N)	67	314	280 (89%)	133 (47.5%)	147 (52.5%)
2011 (N)	50	296	282 (95%)	93 (33.0%)	189 (67.0%)
2001 vs. 2011			#P < 0.01	*P < 0.001	*P < 0.001

^#*^Significantly different between 2001 and 2011, Chi-square test.

## Discussion

This retrospective review of all SICU patients from October to December revealed that there was a significant increase (P < 0.01) in the patients with their serum Mg level measured between 2001 (89%) and 2011 (95%) (Table 1). There was a significant decrease (P < 0.001) in patients with hypomagnesemia (< 2 mg/dL) between 2001 (47.5%) and 2011 (33.0%) (Table 1). On the other hand, there was a significant increase (P < 0.001) in patients with normal serum Mg level (> 2 mg/dL) between 2001 (52.5%) and 2011 (67.0%) (Table 1).

There are several non-mutually exclusive explanations possible for these results. One is the improved understanding of the importance of Mg in hospitalized patients. In the decade since the first sampling, it is possible that Mg is being monitored more regularly in ICU patients. The slightly higher number of samples per patient in 2011 suggests this: The average number of electrolyte determination was 4.69 per patient in 2001 and 5.92 per patient during October to December. One reason for this might be the increased prevalence of ICU fellowship training among practitioners in the ICU.

It is important for ICU practitioners to be aware of hypo-magnesemia since Mg is a cofactor in over 300 enzymatic reactions involving energy metabolism, protein, and nucleic acid synthesis [4]. The membrane pump that creates the electrical gradient across the cell membrane is dependent on Mg. Thus Mg is important in the activity of electrically excitable tissues [10]. Since Mg regulates the movement of calcium in smooth muscle cells, it is important in peripheral vascular tone regulation and in cardiac contractile strength. Because Mg is a cofactor for ATPase reactions, deficiency could lead to defects in cellular energy utilization.

Other electrolyte disturbances often co-exist with hypo-magnesemia. The hypokalemia that accompanies Mg deficiency is often resistant to simply replacing the potassium, and repleting the hypo-magnesemia can be necessary before potassium repletion can be accomplished.

However, because an overall body Mg deficiency may not be accompanied by hypo-magnesemia, the true incidence of Mg depletion is higher than what is detected by the Mg concentration in blood. Since serum Mg levels are not a perfect reflection of total body Mg, it is important to recognize other conditions that can lead to an underlying hypo-magnesemia.

Causes of hypo-magnesemia in the ICU population are diverse. Diuretic therapy is the leading cause of Mg deficiency. Diuretic induced inhibition of sodium resorption will also interfere with Mg resorption. Urinary Mg excretion is most with the loop diuretics (furosemide and ethacrynic acid). Antibiotics which also promote Mg depletion are the aminoglycosides and amphotericin [11]. Antibiotic associated diarrhea can also be accompanied by significant Mg stool losses. Other drugs associated with Mg depletion include digoxin and epinephrine, which shift Mg into cells [12]. In contrast, the chemotherapeutic agents cyclosporine and cisplatin promote renal Mg excretion [12].

Proton pump inhibitors are known to lower serum Mg levels in a variety of patients [13-15]. Their use has increased over the years in question. Since Mg concentration is high in lower gastrointestinal secretions, a secretory diarrhea can lead to Mg depletion [16]. A change in any incidence of prescribing any of the above medications could affect the incidence of hypo-magnesemia observed.

Acute myocardial infarction may be accompanied by hypo-magnesemia in up to 80% of patients in the first 48 h [17]. Chakraborty et al also showed significantly lower Mg levels in patients with acute myocardial infarctions [18]. An et al studied patients younger than 50 years old who had drug eluting stents placed. In patients with subsequent myocardial infarction, after adjusting for age, positive family history, smoking status, hypertension, hypercholesterolemia, and diabetes at baseline, the risk was 8.11-fold higher for patients with quartile 1 of Mg levels than quartile 4 Mg level. In addition, when tested as a continuous variable, serum Mg was a significant predictor for major adverse cardiac events of acute myocardial infarction after adjustment for other confounders [19]. Dibaba et al performed a meta-analysis and systematic review indicating that dietary Mg intake is significantly and inversely associated with serum C-reactive protein levels. They suggest that any potential beneficial effect of Mg intake on chronic diseases may be, at least in part, explained by inhibiting inflammation [20].

Insulin-dependent diabetic patients can exhibit hypo-magnesemia as a result of glycosuria producing urinary Mg losses [21]. Yang et al found that postpartum serum Mg level was also a possible predictor for development of type II DM and that serum Mg level in the postpartum period may be a possible predictor for type II DM development in women with a history of gestational DM [22].

Alcohol use is a very common occurrence in the trauma population. Alcohol is associated with perturbations in Mg levels. Hypo-magnesemia is seen in 30% of hospital admissions for alcohol abuse, and in 85% of delirium tremens admissions [16, 23]. Hypo-magnesemia may be a common laboratory clue towards alcohol abuse in a hospitalized patient who is non-communicative (for example, an unconscious trauma patient, in whom a medical history is unobtainable).

### Study limitations

The incidence of alcohol abuse was not measured in our study, so we cannot comment on the influence of alcohol on our patients’ hypo-magnesemia. Mg concentration is high in lower gastrointestinal secretions, and a secretory diarrhea can lead to Mg depletion [16]. We did not measure the incidence of *Clostridium difficile* (*C. diff*) colitis in the two time periods; however, some institutions report an increased incidence of *C. diff*. In addition, the emergence of the NAP1/BI/O27 strain of *C. diff*. in the early 2000s has been associated with more severe forms of *C. diff*. [24]. Likewise, we were not able to quantitate the incidence of diuretic use which could affect Mg levels. Changing patterns of antibiotic and anti-fungal use could also influence the incidence of hypomagnesemia. Certainly much less amphotericin (which can produce hypo-magnesemia) is being used now than in 2001. Conversely, more proton pump inhibitors, which are known to often decrease Mg levels, were being used in 2011 than in 2001. This study was designed to investigate trends in Mg levels over 10 years in the SICU population. As such, it reflects all Mg levels measured in that 3 month period in the 2 years. We cannot therefore comment on trends in initial Mg levels upon initial admission to the SICU. Also we cannot address the effectiveness of Mg repletion on the results, since all lab values were examined. Likewise, our results reflect our patient population and cannot be generalized to other ICU populations.

### Conclusions

In our tertiary hospital SICU sample, this retrospective review showed there was a significantly lower incidence (P < 0.001) of patients with hypomagnesemia (< 2 mg/dL) between 2001 (47.5%) and 2011 (33.0%). This is encouraging that Mg is more often within normal range a decade later in our SICU population. Possible contributors to this lower incidence include better practitioner training, more frequent laboratory monitoring, and changing patterns of diuretic and amphotericin use.

This retrospective review of all SICU patients from October to December revealed that there was a significant increase (P < 0.01) in patients who had their serum Mg level measured between 2001 (89%) and 2011 (95%). On the other hand, there was a significant increase (P < 0.001) in patients with normal serum Mg level (> 2 mg/dL) between 2001 (52.5%) and 2011 (67.0%). These two findings show that Mg is being measured more often, and being more often maintained in the normal range in 2011 when compared to 2001. Further investigation is needed to better understand these factors.
